# functional mobility tests for evaluation of functionalities in patients with adult spinal deformity

**DOI:** 10.1186/s12891-022-05342-5

**Published:** 2022-04-27

**Authors:** Hyung Rae Lee, Jiwon Park, Dae-Woong Ham, Byung-Taek Kwon, Seong Jun Go, Ho-Joong Kim

**Affiliations:** 1 Department of Orthopedic Surgery, Uijeongbu Eulji Medical Center, University of Eulji College of Medicine, Uijeongbu, Republic of Korea; 2 Department of Orthopedic Surgery, Ansan Korea Medical Center, University of Korea College of Medicine, Ansan, Republic of Korea; 3 Department of Orthopedic Surgery, Choong-Ang University Hospital, Seoul, Republic of Korea; 4 Department of Orthopedic Surgery, Myongji Hospital, Goyang, Republic of Korea; 5 Republic of Korea Army, Seoul, Republic of Korea; 6 Spine Center and Department of Orthopedic Surgery, Seoul National University College of Medicine and Seoul National University Bundang Hospital, 82, Gumi-ro 173 Beon-gil, Bundang-gu, Seongnam, 13620 Gyeonggi-do Republic of Korea

**Keywords:** Adult spinal deformity, Lumbar spinal stenosis, Functional mobility tests, Patient-reported outcomes

## Abstract

**Study design:**

Retrospective cohort study.

**Background:**

Current evaluation of patients with adult spinal deformity (ASD) is mainly based on radiographic parameters derived from X-rays. However, due to their static nature, X-rays fall short of assessing the dynamic functionalities including balance, gait, and the risk of falling. This study aimed to determine the functionalities of ASD patients by measuring functional mobility tests (FMTs) and compared the relationships between patient-reported outcomes (PROs) with FMTs and radiographic parameters to determine whether FMTs are useful evaluation tools for the evaluation of patients with ASD.

**Methods:**

This age- and sex-matched case–control study included 66 patients with ASD and 66 patients with LSS, all of whom were scheduled to undergo spinal surgery. All patients were evaluated with four FMTs including alternate step test (AST), six-meter walk test (SMT), sit-to-stand test (STS), and timed up and go test (TUGT). Correlations of the PROs with FMTs and static radiographic parameters were analyzed.

**Results:**

The baseline characteristics were not significantly different between the two groups. However, compared with patients with LSS, those with ASD showed significantly poorer performance on all four FMTs, spending significantly more time performing the SMT, STS, and TUGT (*P* = 0.046, 0.045, and 0.015, respectively). The results of the four FMTs were significantly correlated with the ODI (Oswestry Disability Index) scores only in the ASD group and not in the LSS group.

**Conclusions:**

FMTs were appropriate tools for assessing the dynamic functionalities of patients with ASD. FMTs might play a bridging role between static radiographic parameters and subjective PROs when treating patients with ASD.

## Background

Adult spinal deformity (ASD) is caused by age-related degenerative changes [1]. Aside from the gross structural malalignment, spines with deformities in the sagittal and coronal plane are also often associated with muscle deteriorations of the spine, pelvis, and lower limbs [2, 3]. These deteriorations negatively affect the coordination of muscles, overall body balance, and normal gait patterns. As such, patients with ASD are at high risk of falls, as the postural balance has been shown to significantly differ between fallers and non-fallers [4–8]. Currently, the evaluation of patients with ASD is mainly based on radiographic parameters derived from X-rays. However, due to their static nature, X-rays show subpar performance in assessing dynamic functionalities including balance, gait, and the risk of falling [9].

Recent studies reported the feasibility of several assessment tools for evaluating the dynamic properties of ASD [10–12]. Functional mobility tests (FMTs) have been validated for the assessment of physical function, trunk and lower limb muscle integrity, and body balance. Accordingly, FMTs have been widely used in patients with different pathologies including old age, hip fractures, knee osteoarthritis, and lumbar stenosis [13–15]. Importantly, FMTs have been established to be useful for revealing the risk of falling [13, 14, 16, 17]. In addition, FMTs are simple, easy to perform, and do not require special equipment. Therefore, FMTs may be useful in preoperatively assessing the functionalities of ASD and anticipating the surgical outcomes.

Lumbar spinal stenosis (LSS) is a spine disease that was reported to show decreased functionality in terms of FMTs [15, 18, 19]. Therefore, in this study, we compared the results of FMTs in patients with ASD and those with LSS in order to evaluate functionalities of ASD patients and derived the correlation between patient-reported outcomes (PROs) with FMTs and radiologic parameters.

## Methods

### Patients

**Fig. 1:**
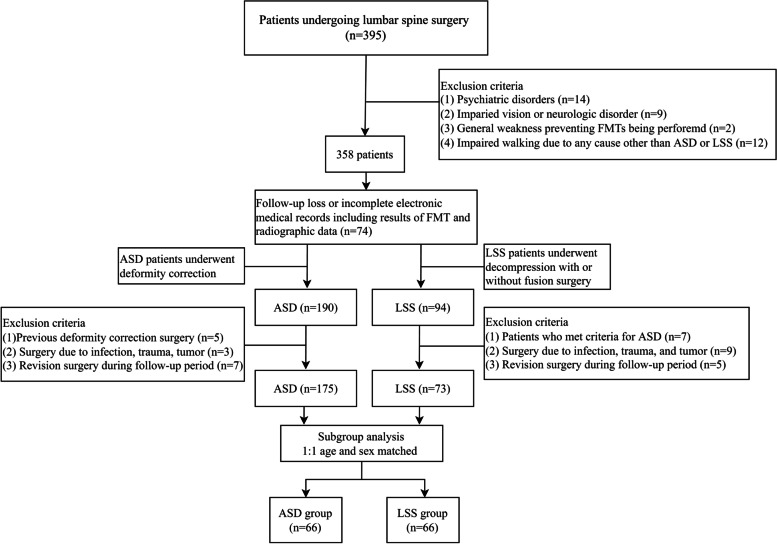
Flowchart of patient selection

This age- and sex-matched case–control study included two groups of patients—one with ASD and the other with LSS—who were scheduled to undergo spinal surgery at our institution between July 2017 to September 2020. We retrospectively analyzed the data of patients who underwent deformity correction surgery with or without decompression for ASD and those who underwent lumbar spine surgery, consisting of decompression with or without fusion for LSS. ASD was defined as a sagittal vertical axis (SVA) > 5 cm, pelvic tilt (PT) > 20°, or pelvic incidence (PI) − lumbar lordosis (LL) > 20 on lateral radiographs in the standing position. Patients were excluded if they had other conditions that influenced their functional performance, such as (1) psychiatric disorders including depression or treatment with sedative drugs; (2) impaired vision or any neurologic disorder which affects physical activities hindering evaluation of functionalities including Parkinson’s disease, epilepsy, and polio; (3) general weakness which prevents FMTs from being performed; or (4) impaired walking due to any cause other than ASD or LSS. Patients were also excluded if their electronic medical records were incomplete as determined by a thorough review of the records and by interviewing the patients. A detailed flowchart of patient selection is shown in Fig. 1. The protocol of this study was approved by the institutional review board of Seoul National University Bundang Hospital (B-1912/580–109).

### Clinical data and FMTs

Preoperative evaluation included demographic factors (e.g., sex, age, height, and weight), tests of handgrip strength (HGS) [19], the Oswestry Disability Index (ODI), scores on the EuroQoL-5D (EQ-5D), and the visual analog scale (VAS) scores for back and leg pain. All patients were evaluated with four previously validated FMTs [12, 20, 21]: the Alternate Step Test (AST), the Six-Meter Walk Test (SMT) [22], the Sit-to-Stand (STS) test, and the Timed Up and Go Test (TUGT) [13, 14, 22]. The AST involves weight shifting and measures the overall stability by evaluating the individual’s ability to maintain balance while standing on one leg during stepping. The SMT measures the time required to walk 6 m at a normal walking speed; the SMT is used to measure the walking ability and speed, with poor walking ability on the SMT being identified as an independent risk factor for recurrent falls. In the STS, patients are asked to rise from a chair of standard height (43 cm) without armrests five times as fast as possible; the results of the STS have been reported to be related to postural control, lower-extremity strength, proprioception, and the risk of falling. In the TUGT, patients are instructed to rise from a standard chair, walk 3 m, turn around, walk back to the chair, and sit down; this test screens for mobility dysfunction and reflects the degree of physical decline and motor-sensory impairment.

### Radiologic measurements

**Fig. 2:**
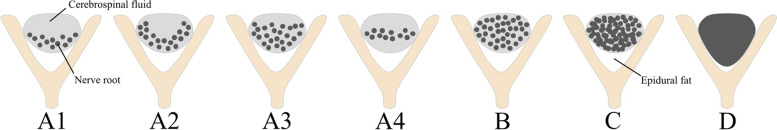
Illustrations showing the morphological classifications of the severity of lumbar spinal stenosis by Schizas et al.

Radiological parameters included (1) sacral slope (SS), (2) pelvic tilt (PT), (3) pelvic incidence (PI), (4) lumbar lordosis (LL), and (5) C7-S1 sagittal vertical axis (SVA). All lumbar levels visible on preoperative T2 axial MRI were evaluated as described in **Fig. ****2** [20]. The degree of stenosis was classified as grade A, B, C, or D according to the relative visibility of cerebrospinal fluid surrounding the rootlets; grade A subgroups were not evaluated. These parameters were measured by two examiners who were blinded to the patient information and not involved in the patients’ treatment.

### Comparison between ASD and LSS groups

The primary outcomes of this study were measurements of four FMTs (AST, SMT, STS, and TUGT) from ASD and LSS groups. The results of four FMTs were compared between the two groups. The secondary outcomes of this study were the relationships between FMTs and PROs. To this, we evaluated PROs including EQ-5D and ODI in ASD and LSS groups, and investigated radiologic parameters. Afterward, the relationship between PROs and FMTs and that between PROs and radiologic parameters were investigated, and the results were compared in each group.

### Statistical analysis

A random sample of 10 patients in each group was selected to establish the test–retest reliability of the four mobility tests, and the test–retest reliability of these four tests was evaluated by calculating their ICCs. Repeated measurements showed high ICCs for the AST (0.88), SMT (0.83), STS (0.87), and TUGT (0.85). The results of these four mobility tests in the ASD and LSS groups were compared by independent *t-*test. Categorical variables including the severity of spinal stenosis were analyzed and compared between the groups using the chi-squared test. The correlations in both groups of ODI and EQ-5D with static radiographic parameters (i.e., SVA, SS, PT, PI, LL, PI-LL) and the four FMTs were analyzed by Pearson’s correlation coefficients. All statistical analyses were performed using the IBM SPSS Statistics for Windows, version 23.0 (IBM Corp., Armonk, NY, USA), with P values < 0.05 defined as statistically significant.

## Results

### Clinical and radiologic evaluation

A total of 132 patients were included in this study. The ASD group (*n* = 66) and the LSS group (*n* = 66) had similar baseline demographic factors including age (73.09 ± 7.60 vs. 71.30 ± 7.72 years), distribution of sex, and weight. The ASD group had a higher BMI and a lower HGS than did the LSS group, albeit the differences were not statistically significant. Clinical parameters, including back pain VAS, leg pain VAS, ODI, and EQ-5D, did not significantly differ between the two groups as well (**Table ****1**). In contrast, the ASD group showed significantly higher values of SVA (*P* < 0.001), PT angle (*P* = 0.006), and PI–LL mismatch (*P* < 0.001) and lower values of SS (*P* = 0.005) and LL (*P* < 0.001) compared with the LLS group. In the simplified Chi-squared test of Schizas grade [20] (A + B) and (C + D) for spinal stenosis, the LSS group showed a significantly more severe spinal canal stenosis than did the ASD group (*P* < 0.001) (**Table ****1**).

**Table 1:** Baseline demographic, clinical, and radiologic characteristics of the adult spinal deformity (ASD) group and the lumbar spinal stenosis (LSS) group

	**ASD group** **(*N* = 66)**	**LSS group** **(*N* = 66)**	***P-*value**
**Age (years)**	73.1 ± 7.6	71.3 ± 7.7	0.35
**Height (cm)**	152.9 ± 8.6	155.8 ± 8.4	0.17
**Weight (kg)**	62.6 ± 10.6	61.9 ± 9.0	0.76
**BMI (kg/cm**^**2**^**)**	26.8 ± 3.9	25.4 ± 2.5	0.10
**M:F, N**	17:49	17:49	-
**Back pain VAS**	6.7 ± 3.0	5.7 ± 2.8	0.19
**Leg pain VAS**	5.8 ± 3.2	6.5 ± 2.3	0.37
**ODI**	23.8 ± 5.5	22.1 ± 5.3	0.19
**EQ-5D**	0.2 ± 0.2	0.2 ± 0.3	0.25
**Handgrip strength (kg)**	27.1 ± 10.9	29.7 ± 12.6	0.42
**Spinopelvic parameters**			
SS angle (º)	24.4 ± 13.2	35.9 ± 12.0	0.005*
PT angle (º)	32.1 ± 13.7	20.9 ± 9.7	0.006*
PI angle (º)	55.5 ± 13.1	56.6 ± 9.9	0.28
LL angle (º)	3.7 ± 28.1	43.2 ± 15.6	< 0.001*
PI–LL angle (º)	52.1 ± 29.6	13.4 ± 16.5	< 0.001*
SVA (mm)	159.3 ± 93.0	17.3 ± 26.1	< 0.001*
**Severity of central spinal canal stenosis, maximum grade, N (%)**			
A	20 (30.3)	5 (7.6)	< 0.001*
B	29 (43.9)	5 (7.6)	
C	14 (21.2)	50 (75.7)	
D	3 (4.5)	6 (9.1)	
Results are reported as mean ± standard deviation (SD) unless indicated otherwise Abbreviations: BMI, body mass index; M, male; F, female; VAS, visual analog scale; ODI, Oswestry Disability Index; EQ-5D, EuroQoL-5D; SS, sacral slope; PT, pelvic tilt; PI, pelvic incidence; LL, lumbar lordosis; SVA, sagittal vertical axis; SD, standard deviation; max, maximum. *P < 0.05			

### FMTs

**Fig. 3:**
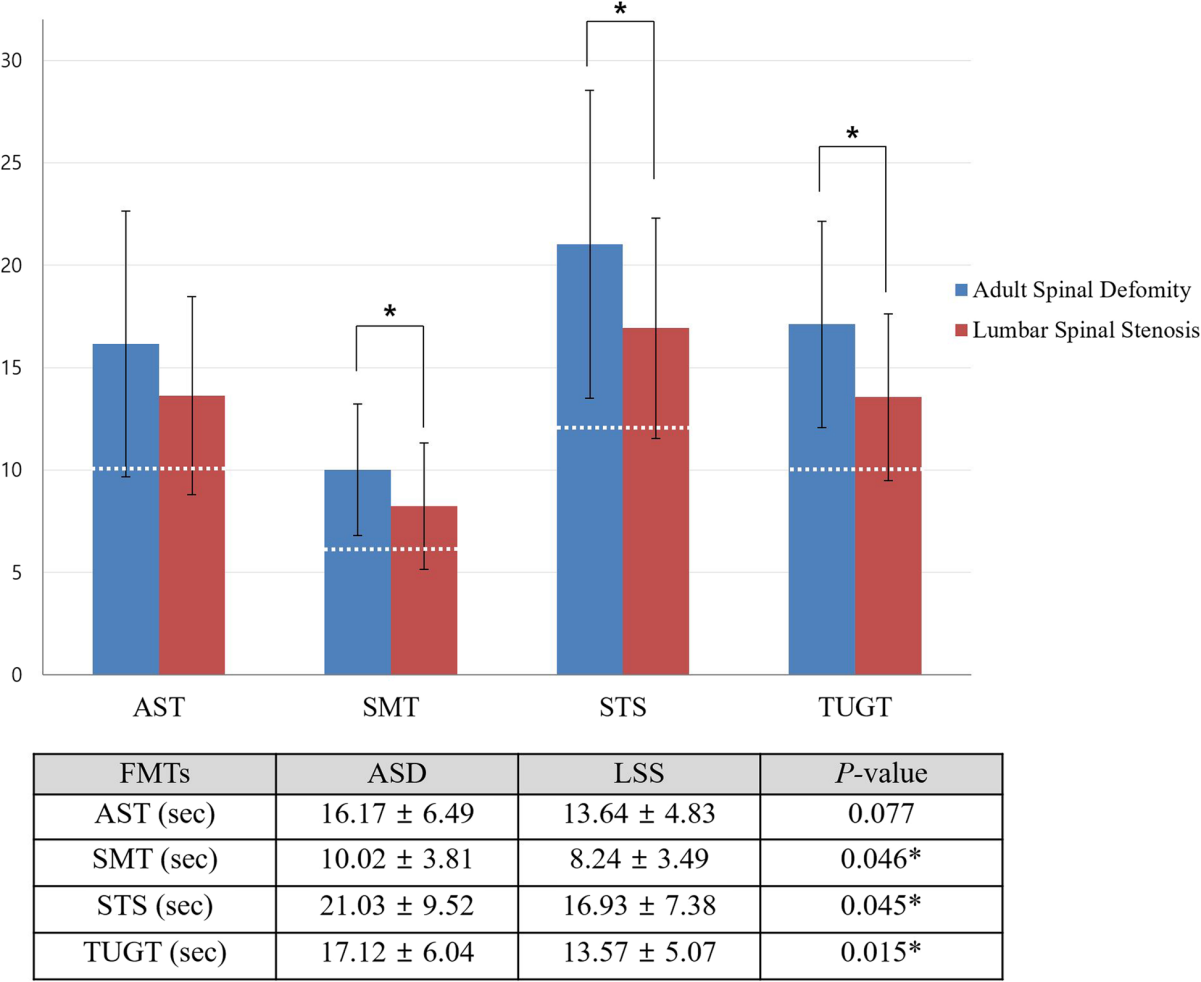
Results of functional mobility tests in patients with adult spinal deformity or lumbar spinal stenosis. White horizontal dotted lines represent the previously suggested cutoff values of each FMT for the risk of falling. *P < 0.05. FMT, functional mobility test; ASD, adult spinal deformity; LSS, lumbar spinal stenosis; AST, alternate step test; SMT, six-meter walk test; STS, sit-to-stand test; TUGT, timed up and go test

Patients in the ASD group showed poorer performance on all four FMTs, spending significantly more time performing the SMT, STS, and TUGT than did those in the LSS group. Patients in the ASD group also showed poorer performance on the AST, but the difference was not statistically significant (*P* = 0.077). More than 95% of patients in the ASD group showed prolonged performance time than the previously validated cutoff time (white dotted line) for the risk of falling (**Fig. ****3**). Repeated measurements of the four mobility tests from random 20 sample patients (ASD group = 10, LSS group = 10) showed high ICCs for the AST (0.88), SMT (0.83), STS (0.87), and TUGT (0.85).

### Correlations of the patient-reported outcomes (ODI and EQ-5D) with FMTs and radiologic parameters

In the ASD group, ODI was significantly correlated with the results of all four FMTs: AST (*R* = 0.344; *P* = 0.049), SMT (*R* = 0.561; *P* = 0.001), STS (*R* = 0.428; *P* = 0.013), and TUGT (*R* = 0.386; *P* = 0.026); moreover, the EQ-5D results was significantly correlated with the results of STS (*R* = -0.396; *P* = 0.023) (**Table ****2**). In contrast, with the exception of the correlation between SVA and EQ-5D (*R* = -0.453; *P* = 0.023), none of the static radiological parameters were significantly correlated with ODI and EQ-5D. In the LSS group, the ODI results were not significantly correlated with any of the results from FMTs or radiologic parameters, while the EQ-5D results were significantly correlated with results of the STS (*R* = -0.522; *P* = 0.002) and TUGT (*R* = -0.348; *P* = 0.047). (**Table ****3**).

**Table 2:** Correlations of ODI and EQ-5D with the results of functional mobility tests and radiologic parameters in the ASD group

		**AST**	**SMT**	**STS**	**TUGT**	**SVA**	**SS**	**PT**	**PI**	**LL**	**PI-LL**
**ODI**	R	0.344	0.561	0.428	0.386	0.374	0.268	-0.085	-0.055	-0.106	0.151
	*P*	0.049*	0.001*	0.013*	0.026*	0.06	0.20	0.67	0.15	0.24	0.50
**EQ-5D**	R	-0.266	-0.253	-0.396	-0.221	-0.453	-0.151	0.054	0.035	0.101	-0.176
	*P*	0.14	0.16	0.023*	0.22	0.022*	0.47	0.80	0.29	0.37	0.16
Abbreviations: ODI, Oswestry Disability Index; EQ-5D, EuroQoL-5D; AST, Alternate Step Test; SMT, Six-Meter Walk Test; STS, Sit-to-Stand test; TUGT, Timed Up and Go Test; SVA, sagittal vertical axis; SS, sacral slope; PT, pelvic tilt; PI, pelvic incidence; LL, lumbar lordosis. *P < 0.05

**Table 3:** Correlations of ODI and EQ-5D with the results of functional mobility tests and radiologic parameters in the LSS group

		**AST**	**SMT**	**STS**	**TUGT**	**SVA**	**SS**	**PT**	**PI**	**LL**	**PI-LL**
**ODI**	R	-0.035	-0.039	0.305	0.113	0.054	-0.157	0.042	-0.109	-0.169	0.105
	*P*	0.85	0.83	0.09	0.53	0.69	0.24	0.76	0.42	0.20	0.43
**EQ-5D**	R	-0.319	-0.228	-0.522	-0.348	-0.055	0.181	-0.055	0.12	0.207	-0.139
	*P*	0.07	0.20	0.002*	0.047*	0.69	0.18	0.68	0.38	0.12	0.30
Abbreviations: ODI, Oswestry Disability Index; EQ-5D, EuroQoL-5D; AST, Alternate Step Test; SMT, Six-Meter Walk Test; STS, Sit-to-Stand test; TUGT, Timed Up and Go Test; SVA, sagittal vertical axis; SS, sacral slope; PT, pelvic tilt; PI, pelvic incidence; LL, lumbar lordosis.*P < 0.05											

## Discussion

In the present study, we found that patients with ASD showed significantly poorer performance in four types of FMTs compared with those with LSS. While the prolonged time of FMTs in the LSS group can be explained by the neurogenic claudication, the poorer results in the ASD group were likely due to a different mechanism. Truncal deformity, which mostly manifests as a positive SVA, induces forward shifting of the center of gravity (CG), affecting the pelvis and eventually inducing deformation in the lower limbs. These deformities in the pelvis and lower limbs may directly induce a compensatory mechanism that manifests as a characteristic crouching gait. This suggests that reduced physical functionalities in ASD are directly reflected in FMTs.

**Fig. 4:**
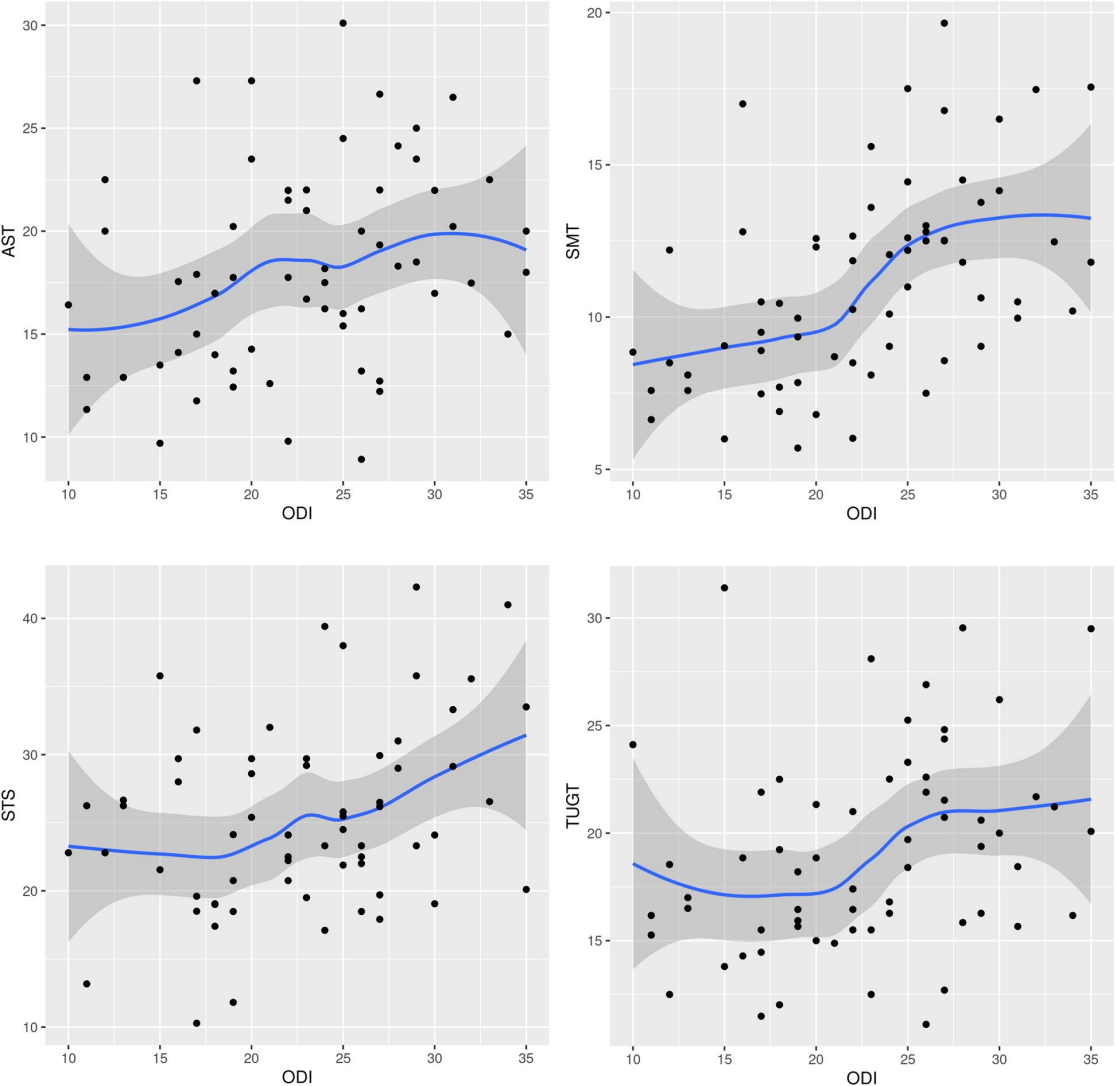
Scatter plots showing the relationship between ODI and four FMTs (AST, SMT, STS, and TUGT). ODI, Oswestry Disability Index; FMT, functional mobility test; AST, alternate step test; SMT, six-meter walk test; STS, sit-to-stand test; TUGT, timed up and go test

**Fig. 5:**
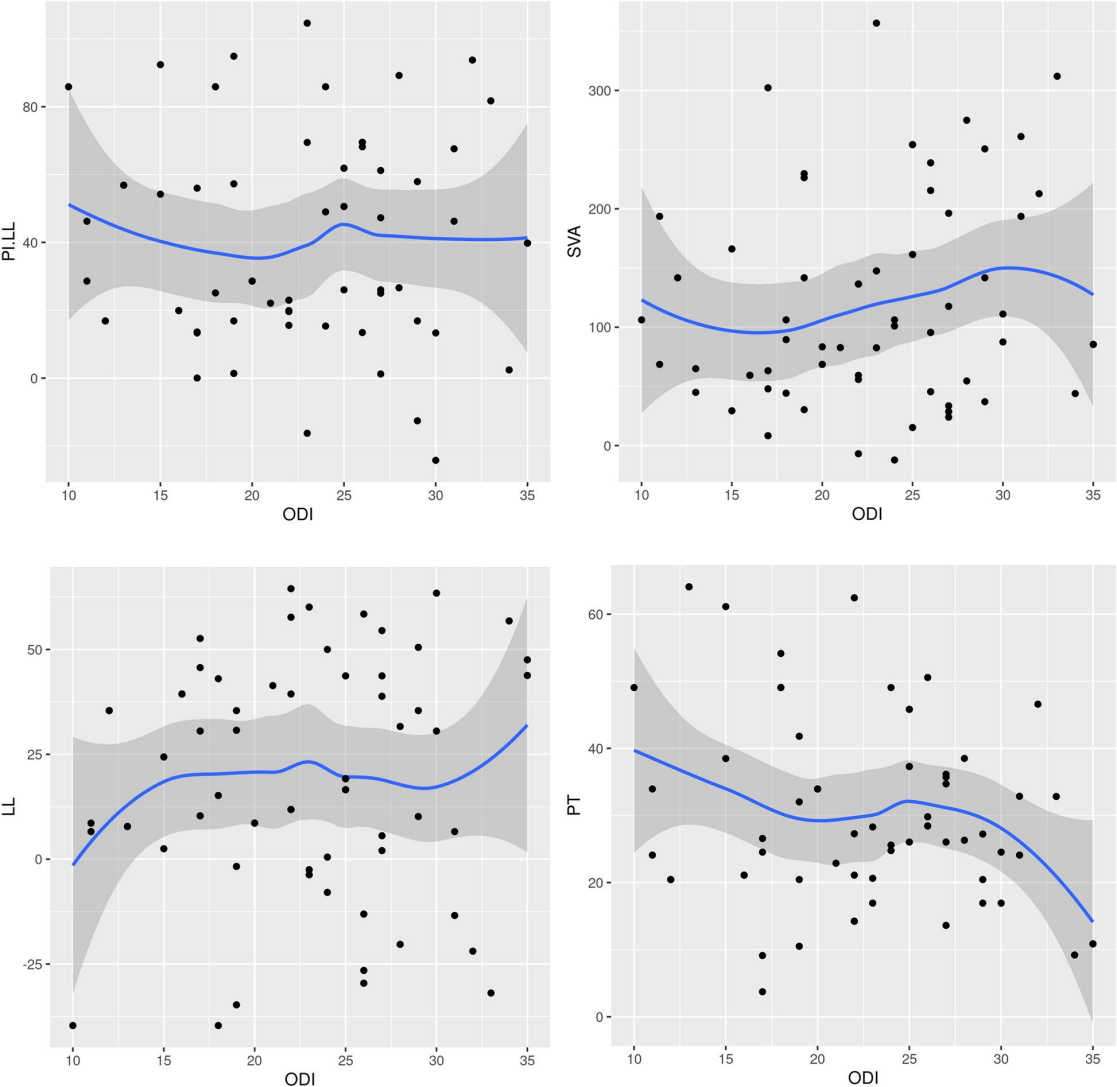
Scatter plots showing the relationship between ODI and radiologic parameters (PI-LL, SVA, LL, and PT). ODI, Oswestry Disability Index; PI, pelvic incidence; LL, lumbar lordosis; SVA, C7-S1 sagittal vertical axis; PT, pelvic tilt

In our study, all four FMTs showed strong correlations with the ODI scores in the ASD group **(**Fig. 4, Table 2**)**. On the other hand, static radiologic parameters did not show significant correlations with the ODI scores (Fig. 5). Although various attempts have been made in terms of the radiographic evaluation of ASD, confirmed standards have not been established [3, 21–24]. In addition, patient-reported outcomes (PROs) can only be described within the limits of their subjective aspects [25]. A recent multicenter, prospective study in ASD patients reported that an SVA of 4 cm or greater was associated with worse ODI, while no strong correlation was discovered between any radiographic parameters and the PROs [9]. Our correlation results in ASD patients add further evidence that FMTs may be useful as an objective evaluation tool for assessing the physical function, trunk and lower limb muscle coordination, overall body balance, and risk of falling in patients with ASD.

Interestingly, only the STS result was correlated with both EQ-5D and ODI of ASD patients and with the EQ-5D of LSS patients. Results of the STS are associated with postural stability, proprioception, and risk of falling [5, 26] as well as the vertical component of CG. Slow movements are required for controlling the CG and maintaining postural stability, especially in physically impaired patients [27]. A forward shift in the CG can cause an individual to fall forward when rising from a seated position. Patients with LSS have difficulty standing in the upright position due to buckling of the ligamentum flavum with truncal extension. On the other hand, patients with ASD have a characteristic anterior stooping posture, which results in an anterior displacement of the CG. The correlation results of STS show that the STS is more sensitive than other FMTs in reflecting the patients’ quality of life. A recent study also revealed that dynamic evaluation using STS was a better predictor for health-related quality of life in patients with ASD [12]. Therefore, physicians aiming to conduct a comprehensive evaluation of ASD including its dynamic aspect may find our results useful, especially regarding STS.

The FMTs have been shown to be sensitive in reflecting the functional impairment in other diseases including hip or knee osteoarthritis and lumbar degenerative disease, and correlated with the PROs of patients with such diseases [15, 18, 28, 29]. Moreover, optimal cutoff points of FMTs have been suggested for identifying patients with a high risk of falling [14, 30]. The suggested cutoff points for the TUGT range from 10 to 16 s, and those for the SMT, STS, and AST have been reported to be 6, 12, and 10 s, respectively [13]. In the current study, the mean values of FMTs of ASD were higher than these cutoff values [15, 18, 19], and also higher than those of LSS (Fig. 3). This suggests that the physical function, body balance, and coordination of muscles in ASD were severely deteriorated to the point of increasing the risk of falling. Patients with LSS were also shown to be at high risk of falling in previous studies [15, 18, 19]. Although ASD shares several characteristics with LSS in terms of stenosis and sagittal deformities, comparison in our study cohort (Table 1) showed significant differences in most of the regional and global sagittal parameters between ASD and LSS. The weak correlation between FMTs and ODI in the LSS group is in agreement with the results of a previous study [15]. The significant relationship of FMTs with ODI scores only in the ASD group suggests that the structural malalignment and deterioration of muscles in the pelvis and lower limbs were more robustly connected with disability in ASD.

Collectively speaking, our results on the FMTs in ASD may change the fashion for treating patients with ASD. A considerable portion of ASD patients with severely deteriorated radiological parameters report that their quality of life is satisfactory [9], which suggests that surgical decisions should not be made solely based on static radiologic parameters. Surgeons need to comprehensively consider multiple factors including subjective complaints of the patients including PROs. Therefore, our results in FMTs might play a bridging role between static radiographic parameters and subjective PROs, which are expected to give useful, adjunctive information to surgeons.

This study has the following limitations. First, we did not investigate the correlations between the results of FMTs and the actual incidence of falling in patients with ASD, because patients underwent surgery immediately after performing the FMTs. However, the actual risk of falling has shown strong correlations with the results of the FMTs [13, 19], and the mean values of these mobility tests were significantly higher in ASD than in LSS patients, in whom the risk of actual falling was demonstrated [19]. Large cross-sectional or longitudinal population-based studies would be helpful for comparing the actual incidence of falling with the results of FMTs. Second, there is a potential for selection bias considering that the ASD group only included patients who were scheduled to undergo surgery at our institution, and not those who were treated with conservative care. Cross-sectional studies that include all patients with ASD are therefore needed, as well as studies assessing whether the mobility function improves after deformity correction surgery and whether improvements in mobility function result in the actual prevention of falls. Third, FMTs are not specific evaluation tools for LSS or ASD. Neurologic claudication is an ischemic symptom that specifically occurs in LSS patients during walking; therefore, it may be questionable whether the impairment due to claudication can be reflected with the six-meter walk test. However, LSS patients experience radiating pain in the lower extremities even when standing with a straight back, and this is particularly common in patients with ligament buckling. In these patients, since it is difficult to stand upright, the performance ability of SMT may show a notable decrease. In fact, in a paper published by our group, LSS evaluated using SMT showed deteriorated performance to the extent of risk of falling [15].

Although this study showed that FMTs were correlated with ODI in ASD patients, thereby suggesting that the usefulness and reliability of the FMTs for evaluation of functionalities in patients with ASD should be further investigated. For instance, a future study direction may include comparing the preoperative FMT results of ASD patients and the follow-up FMT after deformity correction surgery. This will determine how well the FMTs and PROs reflect the improvements in the structural alignment by corrective surgery. However, static radiographic parameters have failed to reflect the functionalities of ASD patients for years, and PROs-correlated dynamic evaluation tools have been gaining popularity [10–12, 31]. As part of such an effort, our study evaluated the functionalities of ASD patients using FMTs, which are relatively simple and convenient, and showed that FMTs were significantly correlated with PROs. There are still many issues to overcome in ASD surgery including proximal junctional kyphosis, sarcopenia, personal care, and lifting [32, 33]. Hence, preoperative measurement of FMTs is potentially useful for the confirmation of functional improvement and risk stratification in patients undergoing spinal surgery.

## Conclusion

Mobility function was significantly more impaired in patients with ASD than in those with LSS, and FMTs were significantly correlated with the ODI scores only in those with ASD. Our results suggest that FMTs are proper evaluation tools for assessing the functionalities of patients with ASD. FMTs may offer additive information on physical function and overall body balance in patients with ASD, which would be valuable for surgeons encountering ASD patients with discrepancies between static radiographic measurements and subjective symptoms.

## Disclosure

The authors report no conflict of interest concerning the materials or methods used in this study or the findings specified in this paper. There was no financial support for this research project.

## Data Availability

The datasets generated and/or analyzed during the current study are not publicly available due to ethical concerns, but are available from the corresponding author on reasonable request.

## Abbreviations

ASD: Adult spinal deformity
LSS: Lumbar spinal stenosis
FMTs: Functional mobility tests
PROs: Patient-reported outcomes
ODI: Oswestry Disability Index
EQ-5D: EuroQoL-5D
VAS: Visual analog scale
AST: Alternate step test
SMT: Six-meter walk test
STS: Sit-to-stand test
TUGT: Timed up and go test
